# Reusing health records from farm animal practices at scale: A potential complementary method of surveillance

**DOI:** 10.1002/vetr.70501

**Published:** 2026-03-20

**Authors:** Beverley Hopkins, Peers Davies, Peter‐John Noble, Anna Bunford‐Davies, April Lawson, Gina Pinchbeck, Ifan Lloyd, Robert Smith, Alan D. Radford

**Affiliations:** ^1^ Wales Veterinary Science Centre Aberystwyth UK; ^2^ Institute of Infection, Veterinary and Ecological Sciences University of Liverpool Liverpool UK; ^3^ Iechyd Da (Gwledig) Aberystwyth UK

## Abstract

**Background:**

Disease in primary care frequently represents a surveillance blind spot, particularly for diseases affecting farm animals.

**Methods:**

Electronic health records (EHRs) were collected from four farm animal veterinary practices in Wales (February 2024‒January 2025) as part of a pilot study. Information collected included species treated, date, owner postcode, products sold and clinical free text. Text mining and topic modelling were used to describe treatments and classify syndromes.

**Results:**

In total, 32,799 records were collected. Antimicrobials were prescribed in 32.6% and 63.8% of cattle and sheep records, respectively. The most frequent antibiotic classes in both species were tetracyclines, macrolides, penicillins and penicillin‒aminoglycoside combinations. There were no recorded category A antimicrobials, and category B antimicrobials were prescribed in only 0.12% and 0.04% of cattle and sheep EHRs, respectively. Text mining and topic modelling seemed efficient methods to identify key syndromes, including mastitis, joint ill, lameness and pneumonia, and how these were treated.

**Limitations:**

Some EHRs described more than one animal with different diagnoses, obfuscating the attribution of treatment to syndrome.

**Conclusion:**

The increasing availability of EHRs at scale and in real‐time represents a complementary opportunity to survey disease and treatment on farms. Text mining methods, including artificial intelligence, could efficiently identify important syndromes and provide novel insight into use of antibacterials.

## INTRODUCTION

Understanding the health of farm animals at the population level is essential to safeguard human health and provides a foundation for targeted interventions to improve animal welfare and productivity. Much of farm animal surveillance currently relies on passive reporting of notifiable diseases by owners and veterinarians alike,^1^ passive surveillance of samples submitted to laboratories,^2^, ^3^ and active surveillance around individual diseases such as bovine tuberculosis.^4^ This leaves a potential gap in our understanding of population‐level disease, namely, what is being seen in primary veterinary care but not being reported to those laboratories that participate in surveillance. In reality, this likely means that the bulk of animal disease seen by veterinary surgeons is not the subject of surveillance, that our understanding of the treatments of farm animals in practice is extremely limited and that changing patterns may go unnoticed. Such gaps are brought into sharp focus by the challenge of antimicrobial resistance (AMR), where there is an imperative to understand not just the quantities of antibiotics used but also the effect of higher and lower use.^5^

Most medical practitioners, including veterinary surgeons, now manage their clinical records digitally. These digitised (electronic) health records (EHRs) represent a surveillance opportunity. In human health, initiatives such as Health Data Research UK have empowered the reuse of EHR data, leading to novel insight across a wide range of human health challenges, including surveillance, where they can provide up‐to‐date and low‐cost data.^6^ In animal health, use of EHRs is perhaps best developed in companion animals where, arguably, digitisation of an individual animal's health record occurred earliest and is most complete.^5^, ^7^, ^8^ These have since been followed by similar programmes for equids. In contrast, the understanding and routine use of EHRs recorded in farm animal practice has lagged behind.

As part of a wider programme of work focusing on understanding and mitigating AMR in Wales, we have been piloting the ethics, logistics and feasibility of collecting EHR data from a sentinel network of farm animal practices in Wales. Here, we describe the data collection process and carry out an exploratory study to test the feasibility of using EHRs for quantifying AMU in cattle and sheep and exploring the association between AMU and specific clinical syndromes requested by the funder.

## MATERIALS AND METHODS

The methods used here are largely based on those currently used to collect data on companion animals and, more recently, horses as part of the SAVSNET^9^ and EVSNET projects at the University of Liverpool. In brief, four farm animal practices in Wales, part of the Iechyd Da group and using the RoboVet (Covetrus) practice management software, were recruited based on convenience. Each practice had to display at least one piece of information to inform their clients of their participation, and opt out options, through their practice website, social media and/or waiting areas. For farm animal species, EHRs were then collected by secure file transfer. Each EHR contains fields for the recorded species, date, treatments (and other items) sold, client postcode and anonymised client ID, and any associated clinical narrative. The nature of the captured data includes both veterinary visits to farms and visits by animal owners to the practice. Owners are able to exclude their animals’ data by opting out through the study's website. Ethical approval for FAVSNET was granted by the University of Liverpool Ethics Committee (RETH001082).

### Cleaning and searching text

Cleaning EHR data are recognised as one of the major challenges to its successful use.^6^ The information on treatments and other items sold was received as 7644 distinct free‐text entries (e.g., second calf‐inject, Anesketin injection). The frequent inclusion of dosage information (e.g., 0.7 and 20 mL Cevaprost) meant there were multiple free‐text entries for most pharmaceutical products. These were mapped to agreed terms using a semi‐automated process; all mappings were confirmed by a domain expert. For pharmaceutical products, where possible, items were mapped to the terms used in the Veterinary Medicines Product database,^10^ which defines a therapeutic group, and in the context of this study, defines antimicrobial, antimicrobial intramammary, antimicrobial intramammary with anti‐inflammatory, antimicrobial with anti‐inflammatory, antimicrobial with anti‐inflammatory and antimycotic, and finally antimicrobial with antimycotic. For pharmaceutical products not listed in the product database (e.g., imported products and repurposed human products), equivalent terms were created. Critical antimicrobials were defined according to the European Medicines Agency as category A (avoid), B (restrict—including fluoroquinolones and third and fourth‐generation cephalosporins and polymyxins), C (caution) and D (prudence).^11^ In this first analysis, products were quantified based on sales and not dose of product sold.

Five syndromes (mastitis, lameness, pneumonia, joint ill and abortion) were chosen, based on funder priorities, to assess the feasibility of classifying clinical data in free‐text clinical narratives by two complementary methods: simple pattern searching and topic modelling. Pattern searching is a supervised method based on regular expressions that allows for the identification of key terms and the exclusion of common negations; for example, retrieving ‘cow has mastitis’ but excluding ‘no signs of mastitis’.

Topic modelling is an unsupervised method. It uses the sentence transformer ‘all‐mpnet‐base‐v2’ to embed narratives (convert to vector representations) using the encode method from that model.^12^ The topic model was generated using the BERTopic package, which reduces the dimensionality of the embeddings and then clusters documents according to vector similarity; finally, using term‐frequency/inverse‐document‐frequency analysis to generate the key terms in the generated clusters or topics.^13^ A minimum cluster size of 53 was used, based on trial and error, to generate a representative collection of topics. BERTopic dimensionality reduction used umap with the following parameters: n_neighbours = 50, n_components = 5, min_dist = 0.0, metric_‘cosine’_low_memory = True and random_state = 42. BERTopic clustering uses hdbscan with min_cluster_size = 53, metric_‘euclidean’_cluster_selection method = ‘eom’ and prediction_data = True. The vectoriser used was the Sklearn package CountVectorizer, excluding English stop words and allowing bigram sizes up to two words. In order to infer topic meaning, topic word content was evaluated using word clouds, generated using the WordCloud module in Python.^14^ Finally, each narrative was assigned its highest probability topic, excluding those where this probability was less than 0.1, where the false positivity rate tends to increase (unpublished observations).

## RESULTS

Data were collected between 1 February 2024 and 31 January 2025, representing the first full year of data collection in this pilot. Records were collected from 2071 clients (holdings), of which 1753 (84.7%) had Welsh postcodes. These included 1291 Welsh clients who had at least one EHR mentioning sheep in the species field and 1121 who had at least one EHR mentioning cattle, equating to approximately 9.2% and 12.0% of sheep and cattle premises in Wales, respectively (based on data for 2021).^15^ Forty‐one percent (*n* = 857) of the 2071 clients kept both sheep and cattle.

In total, 32,799 EHRs were obtained, mostly from cattle (19,224; 58.6%) and sheep (12,356; 37.7%). The number of EHRs per client ranged from 1 to 564 (median 18). These data were collected throughout the year but had a spring predominance, with 31.4% collected between March and May.

### Sales and treatments

In total, 7644 distinct free texts were used to describe 82,393 ‘items’ sold (average 2.5 sales for each of the 32,799 EHRs). These texts covered the full range of items sold, including examination/visit fees (993 texts; 13.0%—used or sold in 9546 EHRs), procedures (1004; 13.1%—used in 7988 EHRs—top three TB test, injection, pregnancy diagnosis), laboratory tests (761; 10.0%—used in 1868 EHRs), items (285; 3.7%—used in 2663 EHRs; top three syringe, needle, lubricant) and pharmaceutical products (2390; 31.3%—used in 21,776 EHRs; top three antimicrobials, nonsteroidal anti‐inflammatory drugs [NSAIDs] and vaccines). In total, 822 (10.7%) texts remained unmapped; these were manually screened to ensure that they did not contain pharmaceutical products.

The most common vaccines used in cattle were against bovine viral diarrhoea (380 records; 1.9% of cattle EHRs), bovine parainfluenza 3/bovine respiratory syncytial virus (322; 1.6%) and infectious bovine rhinotracheitis virus (246; 1.2%). In sheep, the most common vaccines were against Orf virus (243 EHRs; 2.0% of sheep EHRs) and *Toxoplasma gondii* (178 EHRs; 1.4%). For NSAIDs, the most common products sold were based on meloxicam (91.8% of NSAIDs) in both cattle (2573 records; 13.4% of cattle EHRs) and sheep (1611 records; 13.0% of sheep EHRs).

**FIGURE 1:**
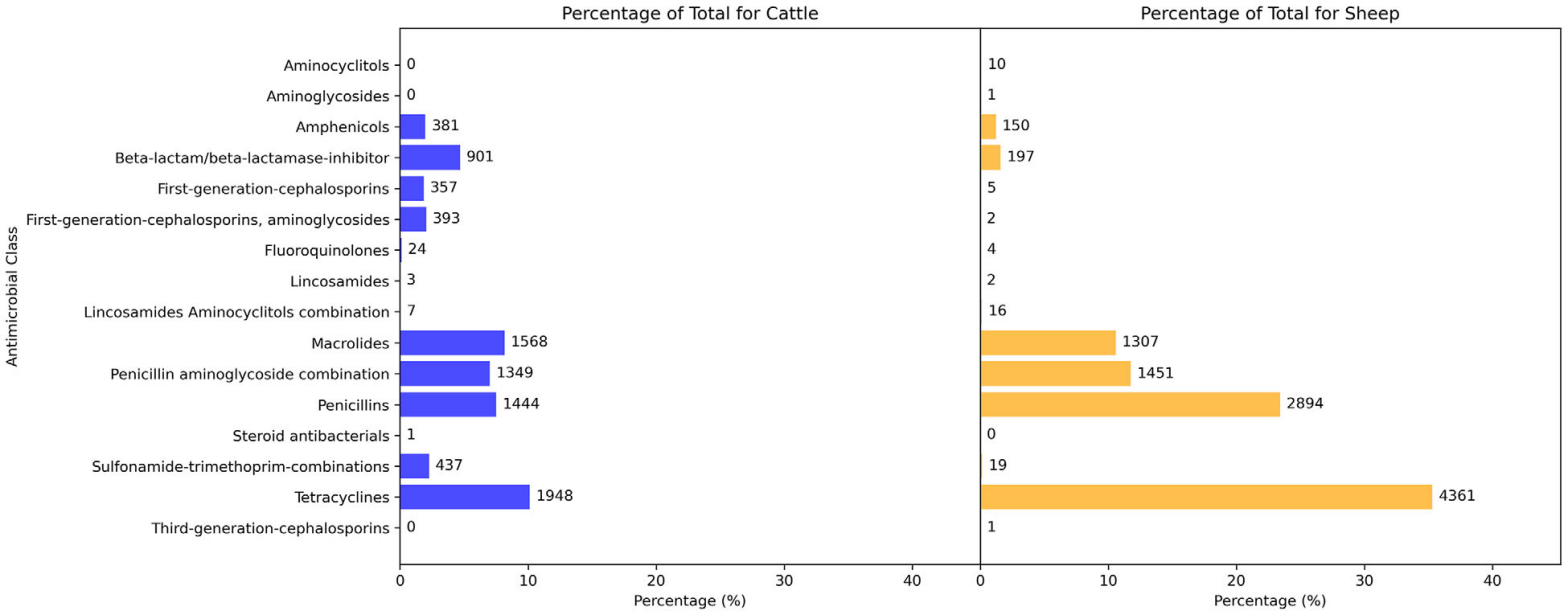
Antimicrobial class use in cattle and sheep records. The denominator used to calculate percentages is total records (cattle 19,224 and sheep 12,356). The given ‘*N*’ is the number of records in which that class was prescribed.

Products containing antimicrobials (including those containing more than one active ingredient) were prescribed in a total of 6495 cattle (32.6%) and 8031 sheep (63.8%) records. The most frequent antibiotic classes in both species were tetracyclines, macrolides, penicillins and penicillin‒aminoglycoside combinations (Figure 1). However, there was a much lower diversity of class used in sheep, where these top four classes made up 95.4% of the prescriptions, compared to 71.6% in cattle.

**FIGURE 2:**
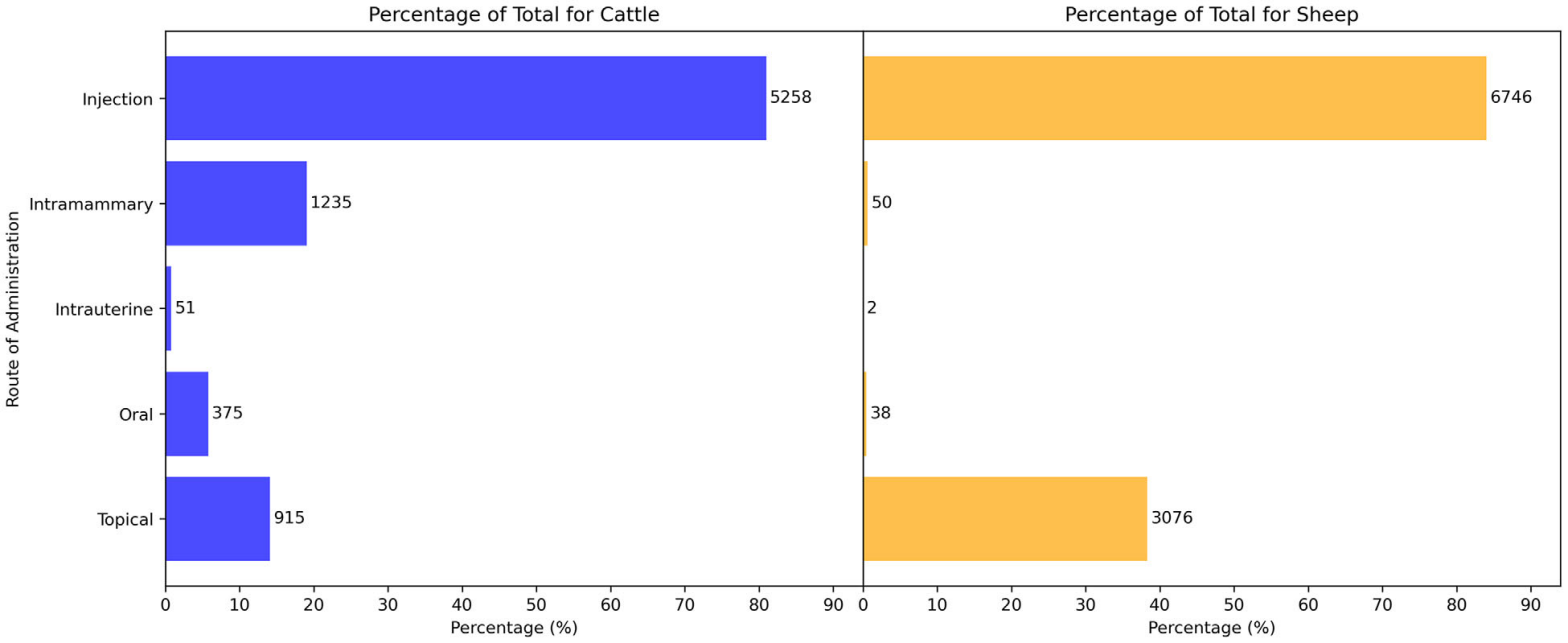
Route of antimicrobial administration in cattle and sheep. The denominator used to calculate percentages was the total number of records in which antimicrobials were prescribed (cattle 6495 and sheep 8031). The given ‘*N*’ is the number of records in which that class was prescribed.

In both species, over 80% of antimicrobial prescriptions were for injectable products. In cattle, this was followed by intramammary treatments, whereas in sheep, it was followed by topical treatment (mostly sprays) (Figure 2).

In cattle, antibiotics were used throughout the year, with a modest rise in spring, such that 30.1% of records recording antibacterial use were in March‒May. In sheep, this spring peak was more pronounced, with 40.1% of antibacterials prescribed March‒April.

Almost all (>99%) of the recorded antibacterials were classified as EMA categories C and D. There were no recorded category A antimicrobials prescribed. Category B antibactierials were prescribed in only 24 (0.12%) cattle and five (0.04%) sheep EHRs and comprised mostly fluoroquinolones used primarily for caesarean sections in cattle and watery mouth in sheep (data not presented). Most of the fluoroquinolone was used in cattle in one participating practice (20 of 4582 cattle EHRs; 0.4%), compared to the other three practices combined (four of 14,642 cattle EHRs; 0.03%).

### Disease

Collecting clinical narratives creates opportunities to explore clinical diseases and to identify why individual antibiotics are used. Of the 32,799 EHRs, 11,959 (36.5%) contained just label information, 9083 (27.7%) were devoid or free text, and clinical free‐text narratives were available for remaining 11,757 (35.8%). These free‐text entries ranged in length from one (only one record) to 3258 characters (median 42). Even the shortest narratives frequently had clinically useful data in them such as ‘PTS’, ‘COD’ and ‘FAWL’). Narratives were further characterised using simple text mining filters based on regular expressions to exclude common negations. The resulting regular expressions returned pneumonia (233 and 120), abortion (74 and 56), joint ill (40 and 108), lameness (266 and 621) and mastitis (135 and 68) associated with EHRs in cattle and sheep, respectively. Manual reading of 100 randomly selected retrieved narratives showed these search terms to have positive predictive values (PPVs) exceeding 90%, apart from abortion, where medically induced abortions were also frequently identified (Table 1). Other reasons for false positives included spelling errors, other more complex forms of negation, and reference to discussions and historical cases (Table 1). In all cases, retrieved records often contained what we termed ‘mixed’ EHRs, where as well as treating the specific syndrome, other animals were also treated for other syndromes. Automated attributing of treatments to syndrome becomes challenging in these mixed EHRs.

**TABLE 1:** Review of 100 random narratives retrieved by regular expression searches for each of five syndromes.

Syndrome	PPV % (mixed %^a^)	False positives	Regular expression (regex)
Mastitis	96^13^	Discuss, historical, vaccine, check for	(?<!no∖ssigns∖sof∖s)(?<!no∖ssign∖s)(?<!no∖ssigns∖s)mastitis *(?!∖svaccine)*
Joint ill	93^9^	Discuss joint ill, no navel/joint ill, no navel or joint ill, never had joint ill/naval ill/scour, label mentions multiple potential uses	(?<!no∖s)(?<!no∖ssign∖sof∖s)(?<!no∖ssigns∖sof∖s)(?<!no∖ssign∖s)(?<!no∖ssigns∖s)*(?<!no∖snavel∖sor∖s)* joint∖sill
Lame	98^17^	Clamed, inflamed	(?<!no∖s)(?<!not∖s)(?<!no∖sobvious∖sswinging∖sof∖sthe∖slegs∖sor∖s)*(?<!in)(?<!c)*(lame)
Pneumonia	98^30^	History of pneumonia, long differential diagnosis list	(?<!no∖s)(?<!no∖ssign∖sof∖s)(?<!no∖ssigns∖sof∖s)(?<!no∖ssign∖s)(?<!no∖ssigns∖s)pneumonia
Abortion	36	23 medical abortions, 9 negations, 29 vaccines	(?<!if∖s)abort *(?!ion∖sva)*

Abbreviation: PPV %, positive predictive value.

^a^ Mixed narratives were those that referred to the treatment of both the syndrome of interest and other animals for other reasons. The original regular expression used to generate the reading set is shown in black. Underlined text indicates the main capture term. Additions to the regex used in the next stages of the project are shown in italics.

**FIGURE 3:**
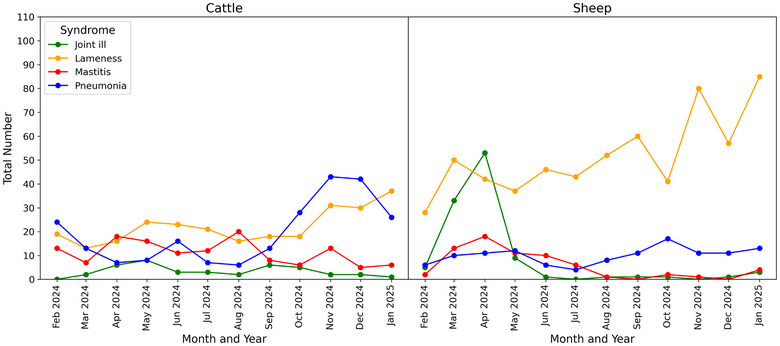
Monthly distribution of four syndromes based on the number of electronic health records identified by text mining in cattle and sheep.

Based on these updated regular expressions, most search terms showed some variability over the 12 months of the study period, with joint ill peaking in March and April, and pneumonia between October and December (Figure 3). Lameness was more evenly distributed throughout the year, although tended to be somewhat lower in the summer. Mastitis had a more complex pattern in cattle, with peaks in April, August and October.

In most syndromes and in both species, the Veterinary Medicines Directorate‐defined therapeutic groups antimicrobials, NSAIDs and corticosteroids were the top three most frequently prescribed medications (Table 2); the exceptions were for mastitis in cattle and sheep (intramammary antimicrobials) and lameness in cattle (local anaesthetic) (Table 2).

**TABLE 2:** Number (percentage) of main treatments prescribed in each of four syndromes (abortion excluded due to low accuracy of regex).

	Pneumonia		Mastitis		Lameness		Joint ill	
Therapeutic group	Cattle	Sheep	Cattle	Sheep	Cattle	Sheep	Cattle	Sheep
Antimicrobial—not IM	186 (79.8)	97 (80.8)	88 (65.2)	60 (88.2)	190 (71.44)	575 (92.6)	32 (80)	95 (88.0)
Antimicrobial—IM	1 (0.4)	0(0.0)	36 (26. 7)	5 (7.4)	2 (0.8)	0 (0.0)	0 (0.0)	0 (0.0)
Anti‐inflammatory corticosteroid	38 (16.3)	12 (10.0)	7 (5.2)	4 (5.9)	11 (4.1)	16 (2.56)	10 (25.0)	60 (55.6)
NSAID	107 (45.9)	43 (35.8)	61 (45.2)	27 (39.7)	114 (42.9)	80 (12.9)	19 (47.5)	18 (16.7)
Local anaesthetic	4 (1.7)	1 (0.8)	2 (1.5)	1 (1.5)	13 (4.9)	1 (0.2)	2 (5.0)	1 (0.9)
Total *N* EHRs	233	120	135	68	266	621	40	108

*Note*: Percentages are calculated using total number of EHRs for the given syndrome.

Abbreviations: EHR, electronic health record; IM, intramammary; NSAID, nonsteroidal anti‐inflammatory drug.

Within each syndrome and between each species, the most common antibacterial class used varied (Table 3). For sheep lameness, tetracycline was most frequent (59.3% of lameness EHRs). Penicillin‒aminoglycoside combinations were the most frequently used for cattle mastitis (22.2%) and sheep joint ill (37.0%). Macrolides were most frequently used for pneumonia in both species and cattle lameness (29.4%‒43.6%). In sheep mastitis, macrolides were joint highest used antibiotic, along with penicillins (both 29.4%). In cattle joint ill, potentiated beta‐lactams were most commonly prescribed (30.0%).

**TABLE 3:** Number (percentage) of records in which antibacterials were prescribed for each of four syndromes.

Antimicrobial class	Pneumonia		Mastitis		Lameness		Joint ill	
	Cattle	Sheep	Cattle	Sheep	Cattle	Sheep	Cattle	Sheep
Amphenicols	48 (20.6)	8 (6.7)	1 (0.7)	1 (1.5)	2 (0.8)	4 (0.6)	9 (22.5)	23 (21.3)
Beta‐lactam/beta‐lactamase inhibitor	7 (3.0)	1 (0.8)	26 (19.3)	6 (8.8)	7 (2.6)	2 (0.3)	12 (30.0)	14 (13.0)
First‐generation cephalosporins	0 (0.0)	0 (0.0)	3 (2.2)	0 (0.0)	1 (0.4)	0 (0.0)	0 (0.0)	0 (0.0)
Lincosamides/aminocyclitols combination	0 (0.0)	0 (0.0)	0 (0.0)	0 (0.0)	0 (0.0)	3 (0.5)	0 (0.0)	0 (0.0)
Macrolides	99 (42.5)	41 (34.2)	18 (13.3)	20 (29.4)	116 (43.6)	137 (22.1)	2 (5.0)	4 (3.7)
Penicillin/aminoglycoside combination	8 (3.4)	4 (3.3)	30 (22.2)	19 (27.9)	25 (9.4)	37 (6.0)	4 (10.0)	40 (37.0)
Penicillins	6 (2.6)	23 (19.2)	21 (15.6)	20 (29.4)	35 (13.2)	207 (33.3)	4 (10.0)	26 (24.1)
Sulphonamide/trimethoprim combinations	4 (1.7)	0 (0.0)	17 (12.6)	0 (0.0)	3 (1.1)	0 (0.0)	0 (0.0)	0 (0.0)
Tetracyclines	47 (20.2)	40 (33.3)	8 (5.9)	17 (25.0)	35 (13.2)	368 (59.3)	5 (12.5)	12 (11.1)
First‐generation cephalosporins/aminoglycosides combination	0 (0.0)	0 (0.0)	13 (9.6)	1 (1.5)	0 (0.00	0 (0.0)	0 (0.0)	0 (0.0)
All antimicrobials	186 (79.8)	97 (80.8)	102 (75.6)	62 (91.2)	190 (71.4)	575 (92.6)	32 (80.0)	95 (88.0)
Total EHRs	233	120	135	68	266	621	40	108

*Note*: Percentages are calculated using the total number of records for the given syndrome.

Abbreviation: EHR, electronic health record.

### Topic models

**FIGURE 4:**
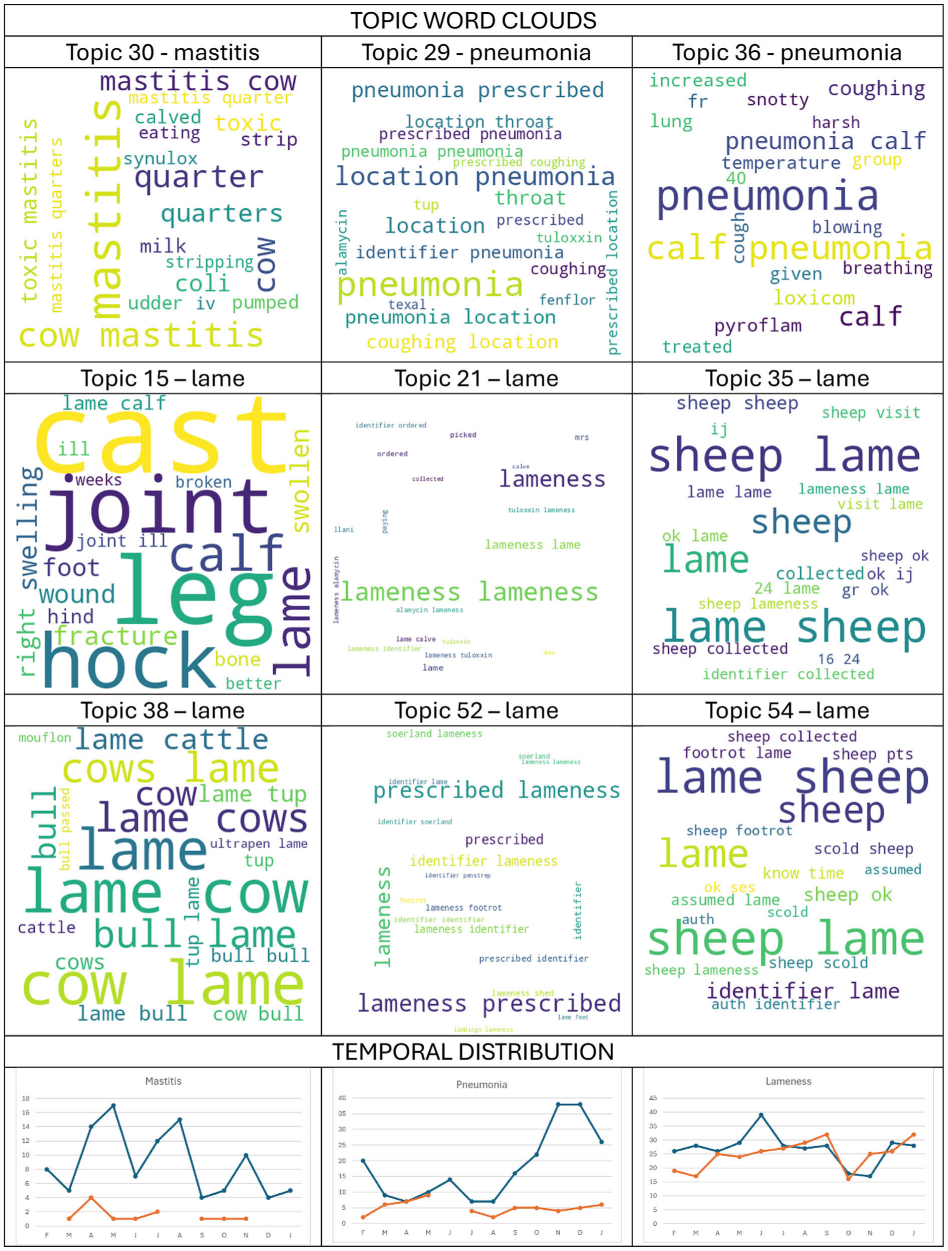
Artificial intelligence‐predicted topics. Top panel—word clouds depicting the important words in each narrative that contribute to each topic and that allow topics to be rapidly assigned to likely syndromes. Bottom panel—temporal distribution for those records most likely to be associated with each topic. Blue = cattle; orange = sheep.

In total, 55 topics were created; based on keywords for the topics, nine closely aligned with three of the chosen syndromes (pneumonia, lameness and mastitis; Figures 4 and S1). Records where these topics were the highest probability of all topics and where the topic probability exceeded 0.1 were manually reviewed and found to be highly accurate for the given syndrome.

Topic 30 (mastitis) returned 118 EHRs (106 from cattle, 12 from sheep; 1.0% of total 11,757 EHR narratives with useable text) and included three false positives (skin infection, metritis, discussion of mastitis control; positive PPV 97.5%). The topic also found three records that would not have been found by the regular expression (two with a narrative of ‘toxic cow gangrenous udder’ and one with ‘cow with mast’). Eight of the mastitis cases also described other non‐mastitic animals being seen for other conditions.

Topics 29 and 36 (pneumonia) returned 269 EHRs (214 cattle and 55 sheep; 2.3% of all narratives). False positives (*n* = 58) were often associated with upper respiratory tract signs, notably laryngeal disease in sheep, giving an overall PPV of 78.4%. Cases captured included spelling mistakes (‘pneumonia’ and ‘pnumonia’) and those where pneumonia was not explicitly mentioned but relevant respiratory signs were (‘blowing’, ‘cough’ and ‘dyspnoea’).

For lameness, nine topics contained the word ‘lame’—manual annotation identified six of particular relevance for lameness (topics 15, 21, 35, 38, 52 and 54) and included 621 EHRs (323 cattle and 298 sheep; 5.3% of narratives). False positives (*n* = 95) were largely associated with non‐specific diseases where locomotory disease was not explicitly mentioned (bloated calf, navel ill, sick calf), giving a PPV of 84.7%. Cases frequently captured narratives where lameness was not explicitly mentioned, including 33 fractures/broken legs, 22 joint ill and one spelling mistake (‘lmae’).

Records found by these three artificial intelligence‐generated topics all showed a broadly similar monthly distribution to that shown for the keyword searching, suggesting that they could form the basis of real‐time surveillance (Figure 4).

## DISCUSSION

The growth of digitised records is creating new opportunities for health research and surveillance in a wide range of species.^6^ However, such opportunities are poorly explored for farmed animals, where EHRs have historically been considered less complete. The aims of this pilot study were to assess the feasibility of collecting data ethically from farm animal veterinary practices, to explore the potential of these data as a future surveillance tool, and to determine whether text mining could be used to describe treatments given at the level of individual syndromes. After deidentification and cleaning, we showed that these data were able to give detailed insight into client interactions including dates, species seen and measures of antibiotic use, such as types of antibiotics used and routes of administration.

One of the major aims of the national AMU strategy is to reduce the use of critical antibiotics in animals. EMA class A critical antibiotics were not identified in this population, and class B were identified in less than 0.2% of records in both species, broadly in line with published sales data in other farmed species.^16^ Most of these class B antibacterials were fluoroquinolones and were used predominantly in one of the four participating practices. The ability to describe therapeutic choices, even those rarely used, is a necessary foundation to better understand their use,^17^ and can have a significant impact on practitioners decisions to use critical antimicrobials.^18^

Use of antimicrobials in this population as measured using EHRs was broadly similar to that shown previously. Elkholly et al. reported that tetracyclines followed by penicillins were the most frequently prescribed by farm animal practitioners in England, with 80% in an injectable form, although their data precluded a breakdown by species.^19^ The high use of injectable penicillin and tetracycline was also shown in data derived from Welsh sheep farms^20^, ^21^ and from farm practices across Great Britain.^22^ In an earlier study of British dairy farms, beta‐lactams (excluding cephalosporins) and aminoglycosides made up most AMU, again by injection.^23^ In our study population consisting of both sheep and cattle, tetracyclines were the most frequently used, followed by penicillins and macrolides, respectively. Whether these differences are due to the passage of time or differences in study population and methodology is unknown. However, the relative ease of using data from EHRs allows for scalable and continuous data collection in near real time, providing the ability to efficiently explore changes over time.^22^ Associated data collected with EHRs can also allow us to describe the species for which products were prescribed, and may therefore complement sales data; the latter provide near to national coverage but may not identify the species of animal for which the product was actually intended.^16^

As well as describing AMU, surveillance of population disease is a vital component of protecting animal industries from disease incursion, and to manage endemic disease. In food animals, there is also an emphasis on protecting human health. In the UK, one of the mainstays of surveillance is through subsidised postmortems, resulting in regular reports of diagnosed disease.^2^, ^3^ Such results are of high accuracy but relatively costly to maintain, and therefore only monitor disease in the likely small proportion of cases generating pathology. As such, most disease on farm may remain under the surveillance radar. Here, we demonstrate the utility of health record data to not only describe the use of key therapeutics but also patterns of disease. We showed that both regular expressions and topic models could accurately identify key syndromes of mastitis, lameness, joint ill and pneumonia; abortion was less accurately identified because of frequent medical induction. Expected seasonal patterns were observed, most notably with sheep mastitis and joint ill peaking in the spring^24^ and cattle pneumonia in winter. In cattle mastitis, although case numbers were not high, peaks in spring, summer and autumn were reminiscent of the varied epidemiology associated with the major mastitis pathogens.^25^ In the future, such tools could provide a necessary foundation for efficient, rapid and scalable population surveillance to spot temporospatial changes and to efficiently create case studies for the identification of risk. Similar methods have been used by the authors to describe an outbreak of severe gastroenteric disease in dogs in 2020,^26^ and by us and others to explore risk factors for disease.^27^, ^28^, ^29^ This continual monitoring approach piloted here can complement more targeted studies where specific hypotheses need addressing.

By combining prescription and narrative data, we have also shown that EHRs can provide additional depth and nuanced understanding of the patterns of therapeutics in general and antibiotic use more specifically, notably in regard to clinical reasoning. We showed that antibiotic use in this population varied across five syndromes and between cattle and sheep. In sheep with lameness, tetracyclines were most frequently used, consistent with other work, where lameness was also shown to be the main driver of antibiotic use in sheep from Northern Ireland.^30^ In contrast, in sheep with joint ill, penicillin aminoglycoside combinations were most frequently used, which contrasted with an earlier questionnaire study of sheep farmers where beta‐lactams were most frequently used.^31^ In the future, by linking to guidelines, these data can then be used to assess the appropriateness of selected antibiotics for a given syndrome.

Studies based on sentinel populations and EHRs, while scalable and efficient, have limitations. Lack of random recruitment means they cannot be considered representative, especially when numbers of practices are low, as in the current study. They also rely on what is recorded in the EHR, both sold (for products) and in the health narrative for disease. Quantification of neither volume of product nor number of treated animals has been considered in this first analysis. Text mining methods can miss cases, such that they should be generally considered an underestimate of actual disease; this limitation would need to be considered if these data were to be used for surveillance. Here, we use the term EHR to describe unique events—how these are divided between visits to the farm and client visits to the practice has not been explored. Quantitative farm animal population metadata frequently used as denominators to allow comparisons in sales data^32^ are not currently recorded on practice management systems. We are therefore currently not able to calculate commonly used farm‐level AMU metrics as the dose of product prescribed is sometimes opaquely recorded and denominator populations are not routinely available. Currently, there is no universal requirement for farms to provide these data to their veterinarian or to calculate these metrics, although a large proportion of farms that participate in voluntary quality assurance schemes do so. In the UK, these metadata are often recorded in a comprehensive and contemporaneous manner on government databases as part of livestock traceability systems. In the future, it would be logical to link these independent data sources to enable detailed farm‐level AMU metric calculations in addition to the AMU pattern recognition from the clinical records. This would also help identify data discrepancies or medicine usage outliers by comparison of these complementary analyses.

In conclusion, we have shown that, through participation and collaboration, it is possible to collect EHR data relating to farm animal health in practice. After cleaning, these data can be used to effectively describe both patterns of disease and treatment. This study provides a necessary foundation to expand disease and therapeutic monitoring routinely on to farm, thereby helping to correct this long‐standing gap in our surveillance efforts. Methods such as text mining and topic modelling offer unique ways to efficiently surface emerging disease trends.

## AUTHOR CONTRIBUTIONS

All the authors helped both develop the academic strategy underpinning the work as well as secure its funding. Bev Hopkins, Anna Bunford‐Davies, Ifan Lloyd and Robert Smith were instrumental in recruiting practices. Peter‐John Noble was primarily responsible for the topic modelling work. Alan D. Radford carried out the regular expression work and with Peers Davies analysed the data and created the first draft of the paper. All the authors were responsible for refining the final submission.

## CONFLICT OF INTEREST STATEMENT

The authors declare they have no conflicts of interest.

## ETHICS STATEMENT

Collection of data for use in this study has been ethically approved by the University of Liverpool Central Ethics Committee (reference RETH001082).

## Supporting information




**Figure S1**. Term frequency inverse document frequency (TFIDF) for the top 20 words in each of nine topics matching important clinical syndromes mastitis (topic 30), pneumonia (topics 29 and 36) and lameness (topics 15, 21, 35, 38, 52 and 54). TFIDF is a measure of the relative importance of each word or bigram in that topic compared to their importance in other topics.

## Data Availability

Health records can contain identifiers and other sensitive information and therefore cannot be made publicly available. Data aggregations may be available on reasonable request to the lead author.
